# Safety and efficacy of ChAdOx1 RVF vaccine against Rift Valley fever in pregnant sheep and goats

**DOI:** 10.1038/s41541-019-0138-0

**Published:** 2019-10-18

**Authors:** Anna Stedman, Daniel Wright, Paul J. Wichgers Schreur, Madeleine H. A. Clark, Adrian V. S. Hill, Sarah C. Gilbert, Michael J. Francis, Lucien van Keulen, Jeroen Kortekaas, Bryan Charleston, George M. Warimwe

**Affiliations:** 1 The Pirbright Institute, Ash Road, Pirbright, Surrey GU24 0NF UK; 2 The Jenner Institute, Nuffield Department of Medicine, University of Oxford, Old Road Campus Research Building, Roosevelt Drive, Oxford, OX3 7DQ UK; 3 Wageningen Bioveterinary Research, Houtribweg 39, 8221 RA Lelystad, The Netherlands; 4 BioVacc Consulting Ltd, The Red House, 10 Market Square, Amersham, HP7 0DQ UK; 5 Laboratory of Virology, Wageningen University, Droevendaalsesteeg 1, 6708 PB Wageningen, The Netherlands; 6 Centre for Tropical Medicine and Global Health, University of Oxford, NDM Research Building, Roosevelt Drive, Oxford, OX3 7FZ UK; 7 KEMRI-Wellcome Trust Research Programme, P.O. Box 230, Kilifi, 80108 Kenya

**Keywords:** Vaccines, Viral infection

## Abstract

Rift Valley fever virus (RVFV) is a zoonotic mosquito-borne virus that was first discovered in Kenya in 1930 and has since spread to become endemic in much of Africa and the Arabian Peninsula. Rift Valley fever (RVF) causes recurrent outbreaks of febrile illness associated with high levels of mortality and poor outcomes during pregnancy—including foetal malformations, spontaneous abortion and stillbirths—in livestock, and associated with miscarriage in humans. No vaccines are available for human use and those licensed for veterinary use have potential drawbacks, including residual virulence that may contraindicate their use in pregnancy. To address this gap, we previously developed a simian adenovirus vectored vaccine, ChAdOx1 RVF, that encodes RVFV envelope glycoproteins. ChAdOx1 RVF is fully protective against RVF in non-pregnant livestock and is also under development for human use. Here, we now demonstrate that when administered to pregnant sheep and goats, ChAdOx1 RVF is safe, elicits high titre RVFV neutralizing antibody, and provides protection against viraemia and foetal loss, although this protection is not as robust for the goats. In addition, we provide a description of RVFV challenge in pregnant goats and contrast this to the pathology observed in pregnant sheep. Together, our data further support the ongoing development of ChAdOx1 RVF vaccine for use in livestock and humans.

## Rift Valley fever: vaccine safety during pregnancy

Rift Valley fever (RVF) causes high levels of foetal loss and abnormality—particularly in sheep and goats. George M. Warimwe and colleagues investigate the safety, efficacy and immunogenicity of the candidate replication-deficient RVF vaccine ChAdOx1 RVF in pregnant sheep and goats. Pregnant animals were vaccinated i.m. during the first trimester and 21 days later challenged i.v. with recombinant live virus to assess protective efficacy. ChAdOx1 RVF is safe in both pregnant sheep and goats with no adverse clinical signs, high titres of neutralizing antibodies and normal pregnancy. ChAdOx1 RVF is strongly protective against subsequent live RVF challenge in pregnant sheep and allows normal foetal development; however, the vaccine is considerably less efficacious in pregnant goats.

## Introduction

Rift Valley fever virus (RVFV) is a zoonotic phlebovirus that is endemic to much of Africa and the Arabian Peninsula.^1,2^ The virus is transmitted by a wide range of mosquito species^3^ and has caused numerous outbreaks since its discovery in Kenya in 1930.^4^ RVFV primarily affects livestock such as sheep, goats, cattle and camels, causing a clinical illness termed Rift Valley fever (RVF) that is characterized by extremely high rates (>90%) of neonatal mortality and abortion in gestating livestock, mainly in sheep and goats.^4^ Human infection mainly occurs through contact with RVFV-contaminated tissues and fluids but can also be transmitted through infectious mosquito bites.^5,6^ In humans, RVF presents as a self-limiting febrile illness that can progress in severity leading to life-threatening complications such as haemorrhagic diatheses and encephalitis with high case fatality rates among hospitalized individuals (>30%) and debilitating sequelae.^7–10^ RVFV can infect human placental tissue,^11^ which may underlie the recently observed association between RVFV infection and spontaneous abortion and stillbirths in pregnant women.^12^

Due to the lack of specific therapeutics, clinical management of RVF is limited to supportive therapy. No vaccines are available for human use and, though licensed veterinary vaccines are available, these have major drawbacks that limit their use.^13^ For instance, the Smithburn vaccine is a highly efficacious live-attenuated RVFV vaccine but is contraindicated in gestating animals as it can result in abortion and foetal malformations.^13–15^ Clone 13, another commercially available livestock vaccine, is based on a naturally attenuated RVFV strain that bears a large deletion in the NSs protein, the main RVFV virulence factor.^16,17^ Whilst Clone 13 is safe and protective after a single dose, an overdose study has shown that Clone 13 can traverse the placental barrier causing foetal malformations and stillbirths.^18^ Formalin-inactivated RVF vaccines can be applied safely during pregnancy but require multiple boosters for optimal efficacy, thus complicating their use in outbreak situations.^13,19^ For humans, only two vaccines have been evaluated in clinical trials: MP-12 and TSI-GSD-200.^20–22^ Both have a good safety profile in humans but MP-12 has been shown to be teratogenic in livestock,^23^ and TSI-GSD-200 requires multiple doses for optimal efficacy.^20^ For these reasons, there is an urgent need for vaccines that provide protection after a single vaccination in animals and/or humans and that can be applied safely during pregnancy.

Following natural RVFV infection, long-lived virus neutralizing antibodies (nAbs) that provide cross-protection against different RVFV strains are generally induced.^24,25^ These nAbs target the conserved viral envelope glycoproteins, Gn and Gc,^26–28^ and are detectable within 1–2 weeks post-infection.^29–31^ We previously developed a candidate vaccine, hereafter termed ChAdOx1 RVF, that is based on a replication-deficient simian adenovirus vector (ChAdOx1) encoding the RVFV Gn and Gc glycoproteins.^32^ Single-dose immunization with ChAdOx1 RVF was shown to safely elicit high nAb titres in sheep, goats, cattle and camels and to provide protection against viral challenge.^33^ The ChAdOx1 RVF vaccine is also under development for human use and may therefore provide a vaccine that can be deployed against the same pathogen in both animals and humans.

Here, to support the use of ChAdOx1 RVF during pregnancy, we conducted a study to examine its safety, immunogenicity and efficacy in pregnant sheep and goats, as these species suffer the greatest burden of mortality, abortion and foetal malformations during RVF outbreaks.^34^ As no RVFV challenge study has previously been conducted in pregnant goats, this study also provides a description of RVFV infection in goats during pregnancy.

## Results

### Vaccine safety in pregnant ewes and does

**Fig. 1:**
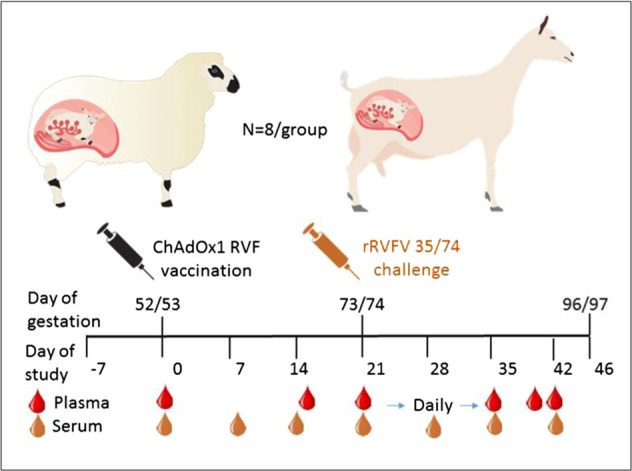
Schematic of experimental design. Inoculation of eight pregnant ewes and goats with 10^9^ infectious units (IU) of ChAdOx1 RVF and eight mock-vaccinated pregnant ewes and goats with PBS at day 52/53 of gestation. The animals were challenged intravenously with 10^5^ TCID_50_ RVFV rec35/74on day 21 post-vaccination (day 73/74 gestation). Plasma (to monitor viraemia) and serum (to measure the antibody response) samples were collected as indicated. At 3 weeks post-challenge, all animals were euthanised, unless reaching humane endpoints prior to this, and the dams and their foetuses examined for signs of abnormalities

Both ewes and does were vaccinated at the beginning of the second trimester at day 52 and day 53 of gestation, respectively (Fig. 1). Animals were vaccinated with 10^9^ infectious units of ChAdOx1 RVF as previous studies with cattle, goats and sheep have shown this dose to be highly effective.^33^ All ewes and does in the ChAdOx1 RVF (*n* = 8/group) and mock-vaccinated groups (*n* = 8/group) were in good health, with no clinical signs or other adverse events following vaccination. ChAdOx1 RVF-vaccinated animals had a slight elevation in temperature of ~0.4 °C in ewes and 0.5 °C in does within 24 h of vaccination. This temperature rise reached statistical significance when compared to baseline (Mann–Whitney *U* test *p* < 0.05 for both species) but had normalized by day 2 post-vaccination. One ewe in the mock-vaccinated group developed laryngeal chondritis (“Texel throat”) unrelated to the study and had to be euthanised 10 days post-vaccination. No other animals showed clinical signs in the 3-week follow-up period before challenge, indicating that the ChAdOx1 RVF vaccine is safe during pregnancy in these livestock species.

### Vaccine immunogenicity and efficacy in pregnant ewes

**Fig. 2:**
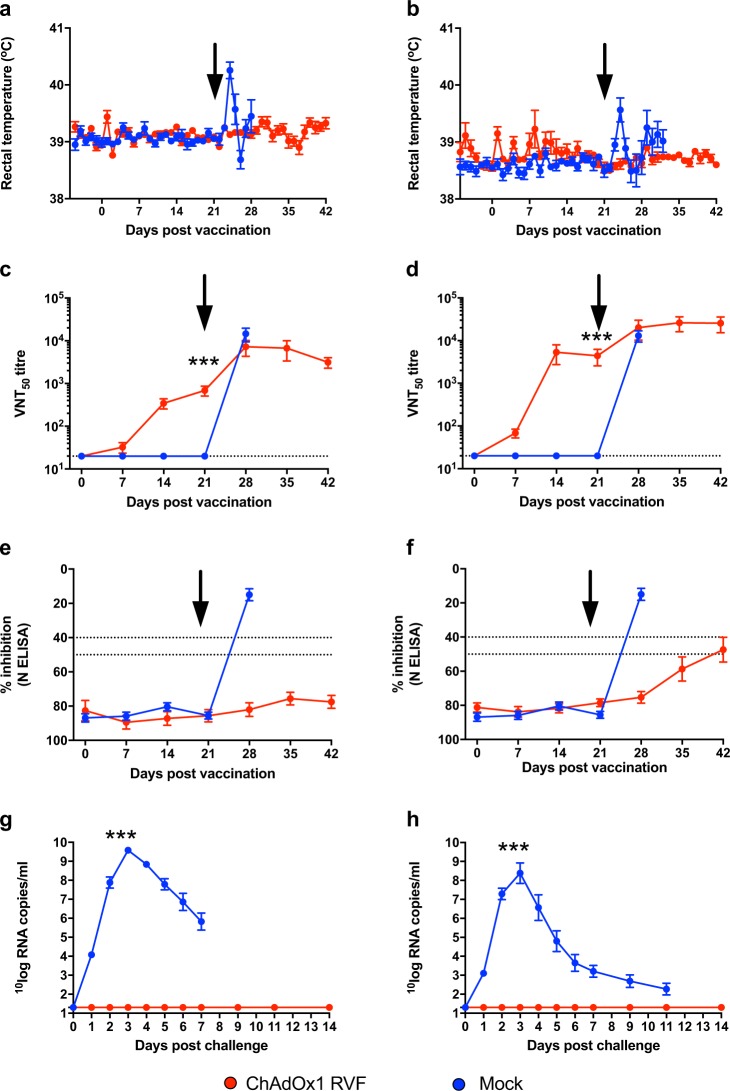
Vaccine immunogenicity and efficacy in pregnant ewes and does. Rectal temperatures, serological responses (RVFV VNT_50_ titres, anti-N ELISA) and viraemia following vaccination and challenge of pregnant ewes and goats are shown. Data in **a**, **c**, **e** and **g** are from pregnant ewes, whilst data in **b**, **d**, **f** and **h** are from pregnant does. Vaccination and challenge were performed as shown in Fig. 1. The black arrows represent the day of challenge. Anti-N ELISA titres (**e** and **f**) are expressed as percentage inhibition calculated as ratio of the optical densities (OD) of the sample and the OD of the negative control (% S/N) as per manufacturer’s instructions. All values lower than 40% are considered positive, between 40% and 50% are considered doubtful and above 50% are considered negative. The dotted lines represent the 40% and 50% inhibition. Samples that tested negative for viraemia are depicted at the detection limit of the RT-qPCR assay (1.3 log10 RNA copies/ml). All data are depicted as means and standard errors. *p* Values from Mann–Whitney *U* test comparing pre-challenge VNT_50_ titres (as measured on day 21; **c** and **d**) and viraemia levels at 3 days post-challenge (**g** and **h**) between mock- and ChAdOx1 RVF-vaccinated animals are shown; ***p < 0.001

**Fig. 3:**
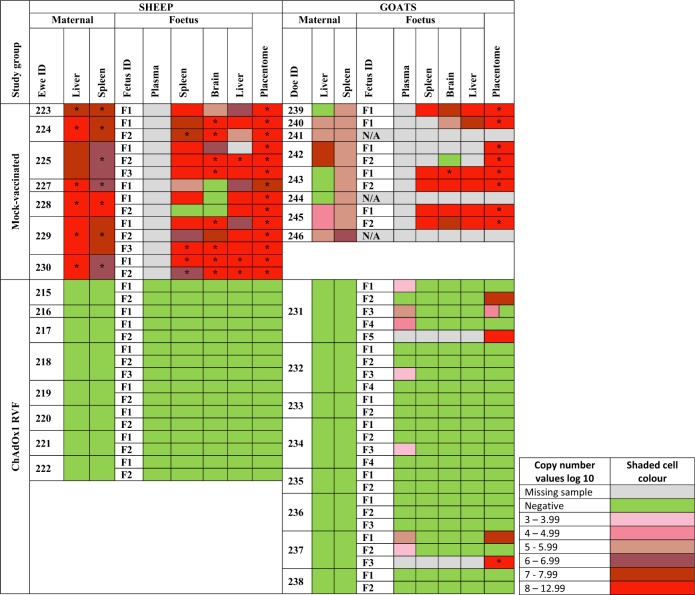
Frequency of viral RNA detection and virus isolation from foetal and maternal tissues. For each species, dam and foetus, results from RT-qPCR detection (detection limit 1.3 log10 RNA copies/ml plasma) and virus isolation (detection limit 2.3 log10 RNA copies/ml 10% organ suspension) are shown post necropsy. The time of necropsy varies between groups (ChAdOx1 RVF 23–24 DPC and mock-vaccinated 8–11 DPC). Placentomes are a combination of maternal and foetal tissue. Samples that were positive by RT-qPCR are colour coded as below, whilst green is used for those that were RT-qPCR negative. Grey shading represents samples that were not available due to autolysis. Samples from which infectious virus could be isolated are marked with an asterisk. Only positive RT-qPCR samples were subjected to viral isolation. N/A (not applicable): does number 241, 244 and 246 were pseudo-pregnant and hence had no foetuses for analysis

**Fig. 4:**
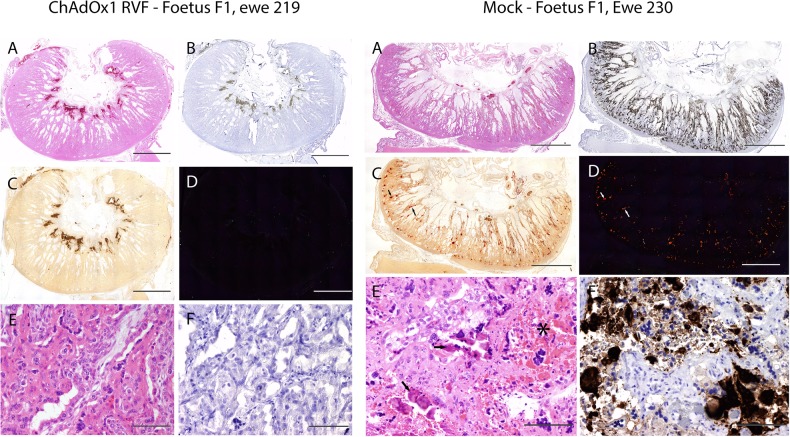
Histopathological analysis of sheep placentomes. Histopathological examination of placentomes of representative sheep from the ChAdOx1 RVF and mock-vaccinated groups are shown. The vaccine allocation and foetus numbers are indicated for each panel; these correspond to foetus and ewe numbers in Fig. 3. For each panel, the letters represent: **a** H&E staining of the placentome, **b** IHC staining with RVFV mAb 4-D4, **c** staining with Alizarin Red showing foci of calcium deposits, **d** same specimen as **c** but visualized under polarized light, **e** high magnification of H&E staining and **f** high magnification of IHC staining. Notice the calcium deposits (arrows in **c** and **d**, right column), haemorrhages and necrotic and calcified epithelium (asterisk and arrows in **e**, right column) in the mock-vaccinated group. Scale bars = 5000 μm (**a**–**d**) and 100 μm (**e** and **f**)

To ensure comparability with previous RVFV vaccination challenge trials with pregnant ewes,^35^ we used a viral challenge dose of 10^5^ TCID_50_. The challenge virus dose was back titrated at 10^5.26^ TCID_50_. Within 3 days after RVFV challenge, mock-vaccinated ewes had elevated rectal temperatures that coincided with a loss of appetite and high levels of viraemia (Figs 2a, g, 3). One ewe died acutely 5 days post-challenge and had a typical necrotic liver and two dead foetuses on necropsy (Supplementary Table 1). Another ewe aborted one foetus 7 days post-challenge together with an autolysed placenta (Supplementary Table 1). At this point, all remaining ewes in this group were euthanised to prevent unnecessary animal discomfort. In total, the mock-vaccinated group carried 14 foetuses, which were all found dead on necropsy (Supplementary Table 1). Viral RNA and infectious virus were detected in maternal livers and spleens and in foetal liver, brain, spleen and placentomes (Fig. 3). Hematoxylin and eosin (H&E) staining of placentomes from mock-vaccinated ewes revealed extensive haemorrhages and areas of necrosis on the maternal epithelium, with foci of mineralization (Fig. 4a, e, right column). RVFV-specific immunohistochemistry (IHC) confirmed the presence of RVFV antigen in both maternal and foetal epithelial cells (Fig. 4b, f right column) and Alizarin Red staining confirmed the presence of calcium deposits (Fig. 4c, d, right column).

In contrast, all ChAdOx1 RVF-vaccinated ewes mounted high titre nAbs against RVFV (Fig. 2c) and showed no clinical signs or viraemia after RVFV challenge (Fig. 2a, g). None of the ewes developed antibodies against RVFV N protein (which is absent in the ChAdOx1 RVF vaccine, but present in the challenge virus) before or after challenge suggesting that the vaccine confers sterile immunity in this species (Fig. 2e). Following euthanasia and necropsy at the end of the study, the ChAdOx1 RVF-vaccinated ewes were found to carry 16 healthy foetuses of the expected size (Supplementary Table 1) with no detectable viral RNA in the maternal organs, blood, foetal organs or placentomes by RT-qPCR (Figs. 2g, 3). IHC staining of the placentomes showed no specific staining for RVFV antigen (Fig. 4b, f, left column). Foci of mineralization were observed in placentomes from some ewes, but these had no impact on the health of the foetuses and no viral RNA could be detected in the placentomes (Fig. 3).

### Vaccine immunogenicity and efficacy in pregnant does

**Fig. 5:**
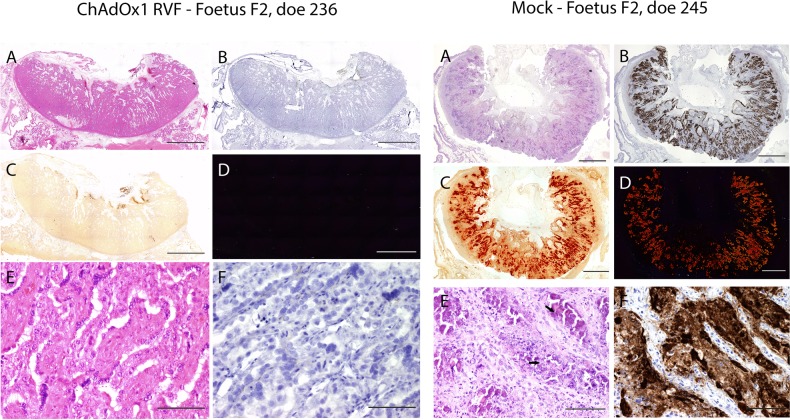
Histopathological analysis of goat placentomes. Histopathological examination of placentomes of representative goats from the ChAdOx1 RVF and mock-vaccinated groups are shown. The vaccine allocation and foetus numbers are indicated for each panel; these correspond to foetus and doe numbers in Fig. 3. For each panel, the letters represent: **a** H&E staining of the placentome, **b** IHC staining with RVFV mAb 4-D4, **c** Staining with Alizarin Red showing foci of calcium deposits, **d** same specimen as **c** but visualized under polarized light, **e** high magnification of H&E staining and **f** high magnification of IHC staining. Notice the calcium deposits (**c** and **d**, right column) and necrotic and calcified epithelium (arrows in **e**, right column) in the mock-vaccinated group. Scale bars = 5000 μm (**a**–**d**) and 100 μm (**e** and **f**)

Following RVFV challenge, all does in the mock-vaccinated group (*n* = 8) showed clinical signs of infection including listlessness, reduced appetite and a transient increase in rectal temperatures starting 2 days post-challenge that was associated with high levels of viraemia (Fig. 2b, h). Two does aborted autolysed foetuses at 8 days post-challenge and, as such, they were culled for reaching a humane endpoint (Supplementary Table 2). The remaining does (*n* = 6) were all euthanised on day 11 post-challenge to prevent unnecessary discomfort. At necropsy, three of the six does were found not to carry foetuses. These does were likely pseudo-pregnant, as confirmed retrospectively by low levels of pregnancy-associated glycoproteins measured by ELISA. All the foetuses in the three remaining pregnant does were dead and autolysed on necropsy (Supplementary Table 2). High viral RNA levels were detected in both maternal and foetal tissues (Fig. 3), but virus could only be isolated, almost exclusively, in the placentomes due to the autolysed status of the foetuses at necropsy (Fig. 3). IHC staining of the placentomes revealed strong staining for RVFV antigen, with large areas of necrotic maternal epithelium and mineralizations observed by H&E and Alizarin Red staining (Fig. 5, right column). However, in strong contrast to mock-vaccinated ewes, extensive haemorrhages were not observed in the placentae of mock-vaccinated does.

All ChAdOx1 RVF-vaccinated does developed RVFV nAbs (Fig. 2d) and remained completely healthy following challenge, with no clinical signs or viraemia (Fig. 2b, h). Three ChAdOx1 RVF-vaccinated does developed antibodies against RVFV N protein after challenge (doe number 234, 237 and 238), suggesting that the challenge virus underwent some replication in the host following infection. Two others (doe numbers 231 and 233) were in the 10% zone between the negative and the positive ELISA values and, as such, were considered doubtful (Fig. 2f). That challenge virus replication occurred, despite the fact that the RVFV nAb titres in does were on average five times higher than those in ewes where such replication did not occur (geometric mean nAb titre at day 21: 2800 in goats vs. 557 in sheep, Mann–Whitney *U* test *p* = 0.001), suggests differences in the mechanisms of protection against RVFV infection between sheep and goats.

At the end of the study all ChAdOx1 RVF-vaccinated does were found to carry a total of 23 apparently healthy foetuses of the expected size and two autolysed foetuses that may have succumbed 1-week post-challenge based on their crown rump lengths (Fig. 3, Supplementary Table 2). The autolysed foetuses were part of multi-foetal pregnancies in two does—one doe carrying five foetuses and another carrying three—but the remaining foetuses carried by these does appeared healthy at necropsy. Organs samples (brain, liver, spleen) of apparently healthy foetuses were additionally assessed for abnormalities. Extensive histological analyses did not reveal any signs of pathology in these samples. None of the maternal tissues for any of the ChAdOx1 RVF-vaccinated does were positive for viral RNA (Fig. 3). However, low levels of viral RNA could be detected in plasma or placentomes from foetuses harvested from four of the eight does and in one exception virus was isolated from a placentome (Fig. 3). The lack of detectable RVFV antigen by immunohistology in the placentomes of any of the live foetuses is explained by the detection limit of the assay. As in all other study groups, foci of mineral deposits were observed in the placentomes of some does (Figs. 3, 5).

## Discussion

We previously demonstrated that ChAdOx1 RVF is safe, highly immunogenic and provides complete protection against RVF in multiple target livestock species.^33,36^ These earlier studies have underpinned the further development of this vaccine in larger ongoing livestock field trials to support registration of the product for veterinary use. ChAdOx1 RVF is also due to enter human phase I clinical trials soon, which will inform the potential use of the same vaccine construct for control of RVF in both livestock and humans. These ongoing and future studies are aimed at addressing the unmet need for a human RVF vaccine, and for safer veterinary RVF vaccine alternatives. However, the safety of the ChAdOx1 RVF vaccine during pregnancy, as well as its immunogenicity and protective efficacy against viral challenge in this physiological state, remained unknown. This study addresses these knowledge gaps by evaluating the safety, immunogenicity and efficacy of ChAdOx1 RVF in sheep and goats, the two main livestock species that bear the brunt of abortion and other poor gestational outcomes during RVF outbreaks.^34^

Pregnant ewes and does immunized with a single dose of ChAdOx1 RVF showed no adverse reactions and remained healthy, with no fever or pregnancy loss in the 3-week post-vaccination period before viral challenge. This was despite the fact that vaccination was performed in the first trimester when the foetus is most susceptible to abortion or malformations following vaccination with current licensed veterinary vaccines.^23^ As expected from previous studies in sheep and goats, all ChAdOx1 RVF vaccinees developed high titre RVFV nAbs and these could be detected as early as 7 days post-vaccination.^33^ As ChAdOx1 RVF does not contain the RVFV nucleoprotein (N), which is present in whole RVFV and widely used in diagnostic ELISAs,^37^ detection of anti-N antibodies can be used to distinguish between infected and vaccinated animals (DIVA). Indeed, all mock-vaccinated animals developed an anti-N antibody response following challenge with RVFV, whilst ChAdOx1 RVF-vaccinated ewes were fully protected from foetal loss and viraemia and did not develop anti-N antibodies. Some of the ChAdOx1 RVF-vaccinated does did develop anti-N antibodies despite the absence of detectable viraemia, suggesting virus replication was not completely blocked. The rapid induction of protective nAbs and together with DIVA compatibility make ChAdOx1 RVF well-suited for response to outbreaks.

ChAdOx1 RVF was fully protective against clinical signs and viraemia in pregnant ewes and does. However, whilst foetuses from ChAdOx1 RVF-vaccinated ewes were free of viral RNA in any of the tissues or plasma at necropsy, those from four of eight ChAdOx1 RVF-vaccinated does had evidence of low levels of viral RNA in plasma or placentomes, with live virus isolated from one of the placentomes. This was despite having an RVFV nAb titre that was on average five times higher than that in sheep on the day of viral challenge. This is remarkable, as the ChAdOx1 RVF vaccine-induced immune response was clearly sufficient to prevent maternal viraemia and clinical signs in this study, and in non-pregnant goats in our previous studies in Kenya.^33^

One possibility is that the immune mechanisms responsible for protection against in utero infection in goats are different from those in sheep. Future studies will help address this conclusively. Such analyses should include correlations between gestational outcomes and challenge virus dose, route of viral exposure, the quality of the humoral and cellular immune response, goat breed and other putative host factors. Non-pregnant goats do indeed show variability in the natural course of RVFV infection depending upon the breed of goat, age, the challenge strain and the route of inoculation.^38–40^ Viral RNA following RVFV challenge has been detected at 28 days post-challenge in non-pregnant goats,^40^ and in spleens of calves and sheep up to 20 days post-challenge, suggesting that animals recovering from RVFV infection may harbour low virus levels in tissues for extended periods. Given that RVFV has tropism for placental tissue, the finding of viral RNA in the placentomes at 23/24 days post-challenge in this study is therefore not surprising.

We observed foci of mineralization (calcium deposits) in placentomes from some animals during necropsy despite the placentae looking healthy macroscopically. These mineralizations were not due to ChAdOx1 RVF vaccination as they were also found in mock-vaccinated ewes and goats and bore no clinical significance. The foci were relatively small compared to the total area of the placentome. Such mineralizations have not been described previously in any of the virulent RVFV challenge studies done in pregnant animals,^41,42^ though the viral challenge in these studies was done later in gestation (second trimester) and vaccinated animals were followed to full term following challenge. The most likely explanation is that the mineralizations were an indicator of inflammation following viral infection as noted for other infectious diseases in animals and humans.^43–45^ The high intravenous challenge dose used in this study would have ensured rapid dissemination of the virus to the placenta and others tissues that RVFV has tropism for, thus initiating a strong host inflammatory response. It is plausible that an alternative inoculation route (e.g. subcutaneous or intranasal or mosquito bite) may have resulted in a slower rate of placental infection with minimal histological evidence of inflammation.^40^ Alternatively, the mineralizations may have been incidental, as occurs during the latter stages of gestation in humans^46,47^ and livestock^45,48^ though its impact on placental physiology is unknown. Future vaccination studies, using different viral challenge routes at different stages of gestation and following pregnancy to full term, will be useful in fully characterizing incidental, transient and irreversible features of placental RVFV infection in livestock.

In summary, we have shown the ChAdOx1 RVF vaccine is safe for use in pregnant sheep and goats, elicits high titre RVFV nAbs, and provides protection against foetal loss. We have also provided a description of the pathology of RVFV infection in pregnant goats and compared this to that in pregnant sheep at the same stage of gestation using the same virus challenge strain, dose and inoculation route. That not all foetuses of ChAdOx1 RVF-vaccinated goats were fully protected, despite having significantly higher RVFV nAb titres than those in ChAdOx1 RVF-vaccinated sheep (whose foetuses were all protected), suggests differences in key mechanisms of protection against foetal infection between the two species. Further studies comparing immune responses in the diverse livestock species affected by RVFV will be required to fully determine the basis of these efficacy differences and may inform development of vaccines to protect pregnant women against RVF.

## Methods

### Ethics statement

Animal experiments were conducted in the Netherlands in accordance with the Dutch Law on Animal Experiments (Wet op de Dierproeven, ID number BWBR0003081) and the European regulations (EU directive 2010/63/EU) on the protection of animals used for scientific purposes. The procedures were approved by the animal ethics committee of Wageningen Bioveterinary Research (WBVR) and the Dutch Central Authority for Scientific Procedures on Animals (permit number AVD401002017816). In addition, the study was reviewed and approved by the Pirbright Institute animal ethics committee.

### Cells and viruses

The ChAdOx1 RVF vaccine encodes the RVFV Gn and Gc coding sequence (GenBank accession number DQ380208) and was constructed by gateway recombination between the ChAdOx1 vector and entry plasmid containing the RVFV Gn and Gc coding sequence.^33,49^ The virulent RVFV rec35/74 strain was used for animal challenge.^35^

### Animal study design

This study was designed to determine the safety, immunogenicity and efficacy of the ChAdOx1 RVF vaccine in pregnant ewes and goats (see Fig. 1 for a schematic of the study plan). The oestrus cycles of Texel cross-breed ewes and Saanen does age >1.5 years were synchronized using progesterone sponges prior to mating with rams or bucks of the same breeds. Thirty-nine to forty-six days later all ewes and does were scanned for pregnancy by ultrasound and 16 gestating animals of each species were selected. The general health of these animals, based on absence of clinical signs of illness, was confirmed by the local veterinarian prior to transporting the animals to the animal facility.

After ~1 week of acclimation, the animals, all on day 52–53 of gestation, were vaccinated intramuscularly in the right brachiocephalicus muscle with either 10^9^ infectious units of the study vaccine ChAdOx1 RVF (*n* = 8) in 1 ml of vaccine diluent (sterile phosphate buffered saline, PBS) or with 1 ml of vaccine diluent for the mock-vaccinated group (*n* = 8). Use of vaccine diluent was an appropriate control since we have never observed protection conferred by an irrelevant adenovirus vector when used in other vaccine studies.^50–52^ To assess protective efficacy, the animals were challenged intravenously with 10^5^ TCID_50_ RVFV rec35/74 (ref. ^35^) on day 21 post-vaccination (day 73/74 of gestation). Animals were monitored daily for general health, rectal temperature and signs of abortion throughout the course of the study. Animals were euthanised at the end of the study (3 weeks post-challenge) or on reaching humane endpoints.

Serum samples were collected weekly throughout the study to measure the antibody responses. Viraemia monitoring was done on plasma collected on the day of challenge (day 21), daily post-challenge for 7 days and thereafter at specified time points. Samples for necropsy were taken from the liver, spleen and placenta of dams, and from the liver, spleen and brain of foetuses. Additional samples were collected from any organs showing abnormalities. All organ samples were placed on ice during the necropsies and subsequently stored at −80 °C. Tissue samples for histology and IHC were collected, placed in formalin, embedded into paraffin and prepared for H&E staining, Alizarin Red (Merck, Darmstadt, Germany) staining for calcium or IHC staining for RVFV antigen using the RVFV Gn-specific 4-D4 monoclonal antibody.^53^

### Laboratory assays

Organ samples, tissue and plasma samples were prepared and tested for the presence of RVFV RNA by RT-qPCR. Viral RNA was isolated with the NucliSens easyMAG system according the manufacturer’s instructions (Biomerieux, France) from either 0.5 ml of plasma or 0.5 ml of 10% organ suspension. Organ sample homogenates were prepared using the ULTRA-TURRAX system in combination with DT-20 tubes (IKA, Staufen, Germany). Briefly, 6 ml of culture medium was added to 0.6 g of tissue. Samples were homogenized for 40 s and cell debris was removed by slow-speed centrifugation. Five microlitresa of the RNA was subsequently used in RT-qPCR using the LightCycler RNA Amplification Kit HybProbe (Roche, Almere, The Netherlands) in combination with a LightCycler 480 real-time PCR system (Roche). One-tube RT-qPCR was performed using the forward primer RVS (AAAGGAACAATGGACTCTGGTCA), reverse primer RVAs (CACTTCTTACTACCATGTCCTCCAAT) and a FAM-labelled probe RVP (AAAGCTTTGATATCTCTCAGTGCCCCAA).^53^ Virus isolation was performed on RT-qPCR positive samples with a threshold above 10^5^ RNA copies/ml as this has been previously shown to be a cut-off point below which no live virus can be detected.^53^ Presence of RVFV nucleoprotein-specific antibodies in sera was determined using the ID Screen® Rift Valley Fever Competition ELISA (ID-Vet, Montpellier, France). Serum RVFV nAbs were measured using a virus neutralization test (VNT). Briefly, in a 96-well plate format, serial dilutions (50 μl) of heat-inactivated sera (2 h, 56 °C) were incubated with 50 μl (103.6 TCID50/ml) of RVFV-4seGFP for 2 h at room temperature. Subsequently, 20,000 BHK-21 cells (in 50 μl) were added to each well. Plates were incubated for 2 days at 37 °C and 5% CO_2_ and scored using an EVOS-FL microscope (Life Technologies). VNT_50_ titres were calculated using the Spearman–Kärber algorithm.^53^

### Statistical analyses

Statistical comparisons between study groups were done using non-parametric tests, with two-sided *p* < 0.05 as the cut-off for statistical significance. All analyses were done with GraphPad Prism 7.

### Reporting summary

Further information on research design is available in the Nature Research Reporting Summary linked to this article.

## Supplementary information

Reporting Summary Checklist

Supplementary Information

## Data Availability

All data generated or analyzed during this study are included in this published article.
