# Supplementary material for: Safety and efficacy of ChAdOx1 RVF vaccine against Rift Valley fever in pregnant sheep and goats
Source: NPJ Vaccines. 2019 Oct 18;4:44. doi: 10.1038/s41541-019-0138-0 (PMC6802222; doi:10.1038/s41541-019-0138-0)
Supplement: Supplementary file 2 — Supplementary Information [file 41541_2019_138_MOESM2_ESM.pdf]

# Supplementary Information

**Supplementary Table 1.** Necropsy findings of the foetuses of mock-vaccinated (**A**) and ChAdOx1 RVF (**B**) vaccinated ewes

## A

| Ewe | Feutus | Weight | crown-tail length | male/female | Date of dissection | Necropsy (DPC) | Gestation day | Remarks fetuses |
|---|---|---|---|---|---|---|---|---|
| 223 | F1 | 377 | 24 | F | 3-12-2018 | 7 | 80 | Dead |
| 224 | F1 | 403 | 21 | F | 3-12-2018 | 7 | 80 | Dead |
| | F2 | 522 | 22 | M | 3-12-2018 | 7 | 80 | Dead |
| 225 | F1 | 360 | 20 | F | 3-12-2018 | 7 | 80 | Dead |
| | F2 | 451 | 21 | M | 3-12-2018 | 7 | 80 | Dead |
| | F3 | 363 | 20 | M | 3-12-2018 | 7 | 80 | Dead |
| 226 | F1 | | | | 14-11-2018 | | | |
| | F2 | | | | | | | |
| | F3 | | | | | | | |
| 227 | F1 | 396 | 21 | F | 3-12-2018 | 7 | 80 | Aborted |
| 228 | F1 | 406 | 21 | M | 1-12-2018 | 5 | 79 | Dead |
| | F2 | 370 | 21 | M | 1-12-2018 | 5 | 79 | Dead |
| 229 | F1 | 425 | 21 | F | 3-12-2018 | 7 | 80 | Dead |
| | F2 | 414 | 19,5 | M | 3-12-2018 | 7 | 80 | Dead |
| | F3 | 498 | 21,5 | F | 3-12-2018 | 7 | 80 | Dead |
| 230 | F1 | 546 | 21,5 | M | 3-12-2018 | 7 | 80 | Dead |
| | F2 | 479 | 21 | M | 3-12-2018 | 7 | 80 | Dead |

## B

| Ewe | Feutus | Weight | crown-tail length | male/female | Date of dissection | Necropsy (DPC) | Gestation day | Remarks fetuses |
|---|---|---|---|---|---|---|---|---|
| 215 | F1 | 1116 | 27 | M | 18-12-2018 | 22 | 96 | Alive |
| | F2 | 1081 | 26,5 | M | 18-12-2018 | 22 | 96 | Alive |
| 216 | F1 | 1119 | 26 | F | 18-12-2018 | 22 | 96 | Alive |
| 217 | F1 | 1144 | 25,5 | M | 18-12-2018 | 22 | 96 | Alive |
| | F2 | 1190 | 25,5 | M | 18-12-2018 | 22 | 96 | Alive |
| 218 | F1 | 1061 | 25,5 | F | 18-12-2018 | 22 | 96 | Alive |
| | F2 | 1075 | 26 | M | 18-12-2018 | 22 | 96 | Alive |
| | F3 | 1087 | 26 | F | 18-12-2018 | 22 | 96 | Alive |
| 219 | F1 | 1124 | 28 | M | 19-12-2018 | 23 | 97 | Alive |
| | F2 | 1208 | 29 | M | 19-12-2018 | 23 | 97 | Alive |
| 220 | F1 | 1132 | 28,5 | F | 19-12-2018 | 23 | 97 | Alive |
| | F2 | 1215 | 27,5 | M | 19-12-2018 | 23 | 97 | Alive |
| 221 | F1 | 1110 | 27,5 | F | 19-12-2018 | 23 | 97 | Alive |
| | F2 | 991 | 26,5 | M | 19-12-2018 | 23 | 97 | Alive |
| 222 | F1 | 1225 | 28,5 | M | 19-12-2018 | 23 | 97 | Alive |
| | F2 | 1073 | 27 | F | 19-12-2018 | 23 | 97 | Alive |

**Supplementary Table 2.** Necropsy findings foetuses of the foetuses of mock-vaccinated (**A**) and ChAdOx1 RVF (**B**) vaccinated goats

**A**

| Goat | Feutus | Weight | crown-tail length | male/female | Date of dissection | Necropsy (DPC) | Gestation day | Remarks fetuses |
|---|---|---|---|---|---|---|---|---|
| 239 | F1 | 331 | 20 | F | 21-12-2018 | 11 | 84 | dead, autolytic |
| 240 | F1 | | 14,5 | | 18-12-2018 | 8 | 81 | aborted, severe autolysis |
| 241 | | | | | 21-12-2018 | 11 | 84 | -- |
| 242 | F1 | | 15 | | 18-12-2018 | 8 | 81 | aborted, severe autolysis |
| | F2 | | 15 | | 18-12-2018 | 8 | 81 | aborted, severe autolysis |
| 243 | F1 | 308 | 20,5 | F | 21-12-2018 | 11 | 85 | dead, autolytic |
| | F2 | 362 | 20,5 | M | 21-12-2018 | 11 | 85 | dead, autolytic |
| 244 | | | | | 21-12-2018 | 11 | 85 | -- |
| 245 | F1 | 46 | 11 | M | 21-12-2018 | 11 | 85 | dead, autolytic |
| | F2 | 40 | 11 | F | 21-12-2018 | 11 | 85 | dead, autolytic |
| 246 | | | | | 21-12-2018 | 11 | 85 | -- |

**B**

| Goat | Feutus | Weight | crown-tail length | male/female | Date of dissection | Necropsy (DPC) | Gestation day | Remarks fetuses |
|---|---|---|---|---|---|---|---|---|
| 231 | F1 | 713 | 26,5 | M | 2-01-2018 | 23 | 96 | Alive |
| | F2 | 727 | 26 | M | 2-01-2018 | 23 | 96 | Alive |
| | F3 | 752 | 27 | M | 2-01-2018 | 23 | 96 | Alive |
| | F4 | 733 | 26 | F | 2-01-2018 | 23 | 96 | Alive |
| | F5 | 170 | 18,5 | F | 2-01-2018 | 23 | 96 | Dead (severe autolysis) |
| 232 | F1 | 657 | 25 | F | 2-01-2018 | 23 | 96 | Alive |
| | F2 | 644 | 25,5 | F | 2-01-2018 | 23 | 96 | Alive |
| | F3 | 667 | 26 | M | 2-01-2018 | 23 | 96 | Alive |
| | F4 | 573 | 24,5 | F | 2-01-2018 | 23 | 96 | Alive |
| 233 | F1 | 669 | 24,5 | M | 2-01-2018 | 23 | 96 | Alive |
| | F2 | 591 | 23,5 | M | 2-01-2018 | 23 | 96 | Alive |
| 234 | F1 | 615 | 26,5 | M | 2-01-2018 | 23 | 96 | Alive |
| | F2 | 682 | 26 | M | 2-01-2018 | 23 | 96 | Alive |
| | F3 | 639 | 24,5 | M | 2-01-2018 | 23 | 96 | Alive |
| | F4 | 623 | 25 | M | 2-01-2018 | 23 | 96 | Alive |
| 235 | F1 | 612 | 25,5 | F | 3-01-2018 | 24 | 97 | Alive |
| | F2 | 747 | 26,5 | M | 3-01-2018 | 24 | 97 | Alive |
| 236 | F1 | 805 | 27 | F | 3-01-2018 | 24 | 97 | Alive |
| | F2 | 488 | 23 | M | 3-01-2018 | 24 | 97 | Alive |
| | F3 | 698 | 24,5 | M | 3-01-2018 | 24 | 97 | Alive |
| 237 | F1 | 702 | 25,5 | M | 3-01-2018 | 24 | 97 | Alive |
| | F2 | 613 | 25,5 | M | 3-01-2018 | 24 | 97 | Alive |
| | F3 | 229 | 18 | M | 3-01-2018 | 24 | 97 | Dead (severe autolysis) |
| 238 | F1 | 682 | 25 | F | 3-01-2018 | 24 | 97 | Alive |
| | F2 | 725 | 26 | F | 3-01-2018 | 24 | 97 | Alive |
